# *QuickStats*: Percentage* of Adults Aged ≥18 Years Who Received an Influenza Vaccination in the Past 12 Months,^†^ by Sex and Age Group — National Health Interview Survey,^§^ United States, 2020

**DOI:** 10.15585/mmwr.mm7045a5

**Published:** 2021-11-12

**Figure:**
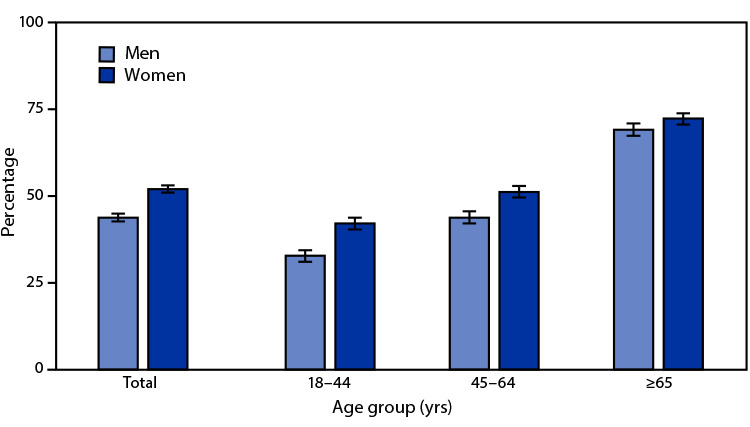
During 2020, 43.7% of men and 51.9% of women aged ≥18 years received an influenza vaccination in the past 12 months, and the prevalence increased with age for both sexes. Among men, 32.7% aged 18–44 years, 43.7% aged 45–64 years, and 69.0% aged ≥65 years received an influenza vaccination. Among women, 42.0% aged 18–44 years, 51.1% aged 45–64 years, and 72.2% aged ≥65 years received an influenza vaccination. For each age group, women were more likely to have received an influenza vaccination compared with men.

**Source:** National Center for Health Statistics, National Health Interview Survey, 2020. https://www.cdc.gov/nchs/nhis/index.htm

